# Lengthening of Insect Development on *Bt* Zone Results in Adult Emergence Asynchrony: Does It Influence the Effectiveness of the High Dose/Refuge Zone Strategy?

**DOI:** 10.3390/toxins4111323

**Published:** 2012-11-15

**Authors:** Aiko Gryspeirt, Jean-Claude Grégoire

**Affiliations:** 1 Biological Control and Spatial Ecology Laboratory (LUBIES), CP 160/12, Université Libre de Bruxelles, av. FD Roosevelt 50, B-1050 Brussels, Belgium; Email: jcgregoi@ulb.ac.be; 2 Fonds pour la Formation à la Recherche dans l’Industrie et l’Agriculture (FRIA), 5 rue d’Egmont, B-1000 Brussels, Belgium

**Keywords:** resistance management, HD/R strategy, emergence asynchrony, random mating disruption, toxin concentration, population pest control

## Abstract

The “High Dose/Refuge” strategy (HD/R) is the currently recommended Insect Resistance Management strategy (IRM) to limit resistance development to *Bacillus thuringiensis* (*Bt*) plants. This strategy requires planting a “refuge zone” composed of non-*Bt* plants suitable for the target insect and in close proximity to a “*Bt* zone” expressing a high toxin concentration. One of the main assumptions is that enough susceptible adults mate with resistant insects. However, previous studies have suggested that the high toxin concentration produced by *Bt* plants induces slower insect development, creating an asynchrony in emergence between the refuge and the *Bt* zone and leading to assortative mating between adults inside each zone. Here, we develop a deterministic model to estimate the impact of toxin concentration, emergence asynchrony and refuge zone size on the effectiveness of the HD/R strategy. We conclude that emergence asynchrony only affects resistance when toxin concentration is high and resistance is recessive. Resistance develops more rapidly and survival of susceptible insects is higher at lower toxin concentration, but in such situations, resistance is insensitive to emergence asynchrony.

## 1. Introduction

Chemical pesticides have been powerful tools for insect pest control for over 4000 years, but they can have numerous negative impacts, such as mortality of nontarget species, and contamination of aquatic and terrestrial environments and human or animal food. To mitigate such negative impacts, alternative methods of pest control have been sought and implemented, including biological control agents, pheromones, insect growth regulators, genetic manipulation of pest species, host-plant resistance, and microbial pest control [1,2,3].

Ninety-five percent of microbial pesticides are composed of *Bacillus thuringiensis* (*Bt*) [4], a Gram positive bacterium producing insecticidal toxins (Cry toxins) during its sporulation [5]. These biological pesticides are specific (harmless for nontarget insects, fish, birds, mammals and humans), are easily produced at industrial scales, and require standard equipment for application. However, *Bt-*based formulations have several limitations: (1) their specificity is a problem if several pests simultaneously attack the same crop; (2) in comparison with synthetic pesticides, the target insect dies later (after one to seven days); (3) toxin persistence is limited in the field (rapid inactivation by environmental factors such as UV, rain and wind) and finally, (4) the *Bt* spray cannot reach mining insects (such as *Ostrinia nubilalis*) [1,6,7].

Genetically modified plants that continuously express a Cry toxin (*Bt* plants) through all tissues circumvent the two last limitations. Since 1996, *Bt* plants have been planted on a global area of about 50 million ha [8]. In 2010, 63% of the corn plants in the United States were transgenic insecticidal hybrids [9]. However, such a constant exposure of large pest populations to *Bt* plants increases the risk of resistance development, which could limit the efficacy of *Bt* plants and, more broadly, the application of effective and authorized microbial pesticides in biological agriculture [6].

To hinder the development of resistant pest populations, several insect resistance management strategies (IRM) have been instigated. The IRM strategy currently most recommended is the High Dose/Refuge zone (HD/R) strategy [10]. This approach requires a “refuge zone,” composed of non-*Bt* plants suitable for the target insect, situated in close proximity to a “*Bt* zone” of high toxin concentration. Susceptible homozygote adults (AsAs) emerge from the refuge zone and resistant homozygotes (ArAr) from the *Bt* zone. If the inheritance of resistance is functionally recessive, the heterozygous progeny (ArAs) produced by mating is killed when ingesting a high toxin concentration in the *Bt* zone at the same rate as homozygous susceptible larvae. The elimination of the heterozygotes greatly slows down the increase of the resistance allele frequency [11]. 

One of the main assumptions of the HD/R strategy is that enough susceptible adults mate with resistant insects. However, studies have shown that Cry toxins significantly slow down immature development in several lepidoptera: *Plodia interpunctella* (Pyralidae) (Hübner) [12,13,14], Pectinophora gossypiella (Gelechiidae) [15,16], *Helicoverpa armigera* (Noctuidae) (Hübner) [17,18], *Ostrinia nubilalis* (Crambidae) [19], and *Helicoverpa zea* (Noctuidae) [20,21]. The slowing down of insect development may induce an asynchrony in emergence between the refuge and the *Bt* zone [13,21,22,23,24], which disrupts mating between resistant and susceptible insects and leads to assortative mating between adults in each of the two zones [15,24,25]. Thus, if resistant males are more likely to mate with resistant females, the proportion of homozygous resistant offspring will be higher than expected with random mating. The reduction of the heterozygous offspring would disturb the effectiveness of the HD/R strategy.

Here, we tested the Cry toxin-induced effect of asynchronous emergency between the refuge zone and the *Bt* zone on the effectiveness of a HD/R strategy. We also assessed the impact of refuge zone size and of Cry toxin concentration on this strategy. For these purposes, we developed a mathematical model integrating several biological parameter values measured in a laboratory colony of *Plodia interpunctella* [13].

## 2. Materials and Methods

### 2.1. Mathematical Model

We used a previously published deterministic and discrete-time simulation model [26] implemented in R (http://www.r-project.org/) and comprising of two parts. The first part is based on population genetics theory and allows for the following of resistance frequency at each generation. Because it is necessary to limit the pest population damaging the crops, the second part is a population dynamics model simulating population density changes. Two kinds of parameters are introduced: operational parameters characterizing the strategy and biological parameters characterizing the target insect. The model and its parameters are detailed in [13]. Each parameter is associated with a default value (see Table 1). 

**Table 1:** Operational and biological parameters introduced in the simulation model: their symbol and default values.

	Symbol	Default Value	Ref.
Operational Parameters			
Refuge zone relative size	*v*	0.05	[27]
Mortality of AsAs on the toxin A (AsAs mortality)	*sBt*	1	[27]
Field area (hectare)		260	[28]
Plants/hectare		67,000	[28]
Biological Parameters			
Initial Ar frequency	*ArFreq*	1.5 × 10^−3 ^	[29]
Ar dominance	*hAr*	0	[30,31]
Fitness cost associated to Ar	*fcost*	0.15	[32]
Fitness cost dominance associated to Ar	*hfc*	0	[32]
% adults emerging from the refuge before the adults of the *Bt* zone, unavailable for random mating	%RefBef	0	[27]
% adults emerging from the refuge simultaneously with adults emerging from the *Bt* zone, available for random mating	%RefRandom	1–%RefBef	
% adults emerging from the *Bt* zone after the adults of the refuge, unavailable for random mating	% *Bt*After	0	[27]
% adults emerging from the *Bt* zone simultaneously with adults emerging from the refuge, available for random mating	% *Bt*Random	1–% *Bt*After	
Initial individual number/ha	*nzero*	50,000	[28]
Intrinsic growth rate	*r*	0.15	[18,33]
Carrying capacity/plant	*K*	22	[28]

**Figure 1:**
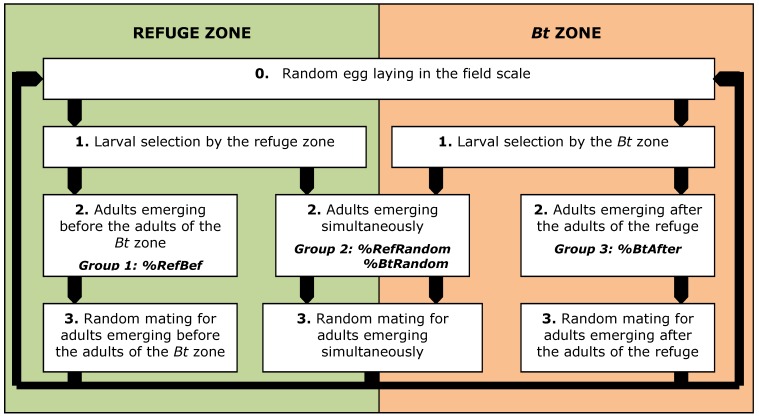
Life stages within one generation, included in the model.

The system considered is a section of a homogeneous, closed, 260ha area [28] composed of two adjacent zones: a *Bt* zone and an unsprayed refuge zone characterized by *v*, its relative size in relation to the global field. The insect population is uniformly distributed between the zones and cannot leave the system. Resistance is an autosomal trait controlled by a single locus with two alleles: the resistance allele Ar and the susceptible allele As. *ArFreq*, the initial frequency of the resistance allele is set to 1.5 × 10^−3^, with no sex linkage and no maternal effect [30,31,34]. Each generation is discrete, continuous (*i.e.*, no diapause) and divided into a succession of simple stages. The generations do not overlap (see Figure 1).

After random oviposition and egg hatching, the larvae are subjected to the selective pressure of the plants they eat. Larval mortality, *sBt*, is linked in our model to toxin concentration in the plants and is dependent on larval genotype (*ArAr, ArAs, AsAs*) and on the dominance of the resistance allele (*hAr*). Insect survival is also influenced by the fitness cost associated with the resistance allele (*fcost*) and its dominance (*hfc*). The fitness cost is defined as a trade-off in which alleles conferring higher fitness in one environment (e.g., presence of *Bt*) reduce fitness in an alternative environment e.g., absence of *Bt*). In our situation, there is a *Bt*-resistance fitness costs in the absence of *Bt* toxins (fitness is lower for resistant insects than for susceptible insects) [35].

In this paper, the fitness of an insect is its ability to survive in relation to its genotype and zone of origin. It is calculated following the Lenormand equations [36] (see Equation 1, Table 2). The function *g*(*x*) equals 1 if the insect is on *Bt* plants and equals zero otherwise. 


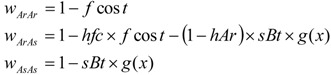
(1)

**Table 2:** Relative magnitude of insect survival (fitness) in relation to zone of origin and insect genotype.

		*Bt* Zone			Refuge Zone		
*hAr*	*sBt*	*ArAr*	*ArAs*	*AsAs*	*ArAr*	*ArAs*	*AsAs*
0	1	0.85	0	0	0.85	1	1
0.53	0.5	0.85	0.765	0.5	0.85	1	1
0.23	0.93	0.85	0.2839	0.07	0.85	1	1

Depending on emergence asynchrony, three different groups of adults are identified [13]. The first two groups are isolated by emergence asynchrony: one is composed of the adults which emerge from the refuge zone before the adults of the *Bt* zone (%RefBef) and the other one is composed of the adults emerging from the *Bt* zone after the adults of the refuge zone (%*Bt*After). These insects are unavailable for random mating between zones. The third group is composed by adults emerging simultaneously from the refuge zone and the *Bt* zone (%RefRandom, %*Bt*Random) and available for random mating between the two zones. Mating is panmictic inside each group and restores the Hardy–Weinberg genotype proportions [37]. 

### 2.2. Model Insect and Experimental Values Introduced in the Model

*Plodia interpunctella*, (Lepidoptera: Pyralidae), our model insect, is a common pest of stored products biologically close to the common pest of *Bt* maize: *Ostrinia nubilalis*,and very easy to culture under controlled conditions in the laboratory. In the model parameters outlined below, the values for insect mortality and for the amount of asynchrony are derived from observed correlations with toxin concentration in *Bt* plants [13]. 

#### 2.2.1. Insect Mortality in Relation to Toxin Concentration in the *Bt* Plant

According to our experimental data [13], a highly significant linear regression equation characterizes, for each toxin, the relationship between mortality before adult emergence and toxin concentration in *Bt* plant [Cry1Ab: *R*^2^ = 83.04 (*F*_1,7_ = 34.29; *p* < 0.001); Cry1Fa: *R*^2^ = 97.96 (*F*_1,7_ = 336; *p* < 0.001)] (see Equations 2 and 3).

Insect mortality (*y*) in relation to toxin concentration, 

*y*_Cry1Ab_ = 403.07*x* − 7.41, *R*^2^ = 83.04

with*x*: µg Cry1Ab/gfresh weight tissue (fwt) in the grain (2)

*y*_Cry1Fa_* =* 0.91*x* − 1.70, *R*^2^ = 97.96

with *x*: ng Cry1Fa/mg total protein (3)

The susceptible insects (AsAs) had 100% mortality at 0.27 µg Cry1Ab/g fwt, 93% mortality at 0.25 µg Cry1Ab/g and 50% mortality at 0.14 µg Cry1Ab/g.

We do not consider mortality at the highest Cry1Fa concentrations because it was extrapolated outside the experimental limits. The AsAs insects had 50% mortality at 57 ng Cry1Fa/mg total protein.

#### 2.2.2. Proportion of Asynchrony in Relation to Toxin Concentration in the *Bt* Plant

We experimentally showed [13] that ingesting a Cry toxin slows insect development. There is a significant linear regression between toxin concentration (Cry1Ab or Cry1Fa) and emergence asynchrony (see Equations 4–7).

Cry1Ab: *Z_Bt_* = 1 − (−418.70*x* + 113.70), *R*²: 0.828

*F*_1,5_ = 24.1; *p* = 0.004**(4)

*Z*_Ref_ = 1 − (−542.3*x* + 122.1), *R*²: 0.763

*F*_1,5_ = 16.09; *p* = 0.012 (5)

Cry1Fa: Z*_Bt_*= 1 − (−1.104*x* + 85.451), *R*²: 0.875

*F*_1,7_ = 49.11; *p* < 0.001 (6)

*Z*_Ref_ = 1 − (−1.14*x* + 91.06), *R*²: 0.917

*F*_1,7_ = 77.94; *p* < 0.001 (7)

Equations 4 and 5: *x* = µg Cry1Ab/g fwt; 

Equations 6 and 7: *x* = ng Cry1Fa/mg total protein*.*

*Z_Bt_*: proportion of adults emerging from the *Bt* zone after all the adults of the refuge zone have emerged. They are unavailable for the random mating between the two zones. *Z_Bt_* is dependent upon the toxin concentration in the *Bt* zone.

*Z*_Ref_: proportion of adults emerging from the refuge zone before all of the adults of the *Bt* zone in relation to toxin concentration in the *Bt* zone. They are unavailable for the random mating between the two zones.

At 0.27 and 0.25 μg Cry1Ab/g fwt, adult emergence is totally asynchronous (%RefBef = %*Bt*After = 100%). At 0.14 μg Cry1Ab/g fwt, 45% of the adults from the *Bt* zone emerge after the adults of the refuge zone (%*Bt*After) and 54% of the adults from the refuge zone emerge before the adults of the *Bt* zone (%RefBef) (see Table 3).

Finally, at 57 ng Cry1Fa/mg total protein, 74% of the adults of the refuge zone emerge before the adults of the *Bt* zone (%RefBef) and 78% of the adults of the *Bt* zone emerge after the adult of the refuge zone (%*Bt*After).

### 2.3. Impact of Emergence Asynchrony (%RefBef–%*Bt*After) on the Effectiveness of Resistance Management

Following the HD/R initial assumptions, all of the adults simultaneously emerged from *Bt* plants expressing a high toxin concentration and from the non-*Bt* zone (see Control in Table 3; s*Bt* = 1, hAr = 0, 0%*Bt*After − 100%*Bt*Random, 0%RefBef − 100%RefRandom). We varied the values of the emergence asynchrony in our simulations (%*Bt*Random and %RefRandom: 0 to 100%) to assess its general impact on the HD/R strategy effectiveness (see Test 1 and Test 2 in Table 3).

Finally, we integrated our experimental data regarding emergence asynchrony and mortality in *Plodia interpunctella* when *Bt* plants produce 0.27, 0.25 and 0.14 μg Cry1Ab/g fwt and 57 ng Cry1Fa/mg total (see Test 3 and Test 4 in Table 3).

**Table 3:** Resistance spread and population control in the population in relation to emergence asynchrony, mortality and resistance dominance.

**A**. *sBt* **1**-*hAr***0**					
	Control	Test 1	Test 2	Test 3	Test 4
Toxin concentration	-	-	-	0.27 µg Cry1Ab/g fwt	112 ng Cry1Fa/mg total protein
%RefRandom	100	10	100	0	-
%*Bt*Random	100	100	90	0	-
GF50Bef (adults from the refuge)	-	45	-	6	-
GF50Random (adults from the refuge and the *Bt* zones)	47	43	13	-	-
GF50After (adults from the *Bt* zone)	-	-	1	1	-
Global GF50	48	45	14	6	-
%popdecrease (between generation 3 and 1)	99.7	99.7	99.7	99.7	-
**B.** *sBt***0.93**-*hAr***0.23**					
	Control	Test 1	Test 2	Test 3	Test 4
Toxin concentration	-	-	-	0.25 µg Cry1Ab/g fwt	104 ng Cry1Fa/mg total protein
%RefRandom	100	10	100	0	-
%*Bt*Random	100	100	90	0	-
GF50Bef	-	7	-	7	-
GF50Random	6	6	7	6	-
GF50After	-	-	6	6	-
Global GF50	7	7	7	7	-
%popdecrease (between generation 3 and 1)	98	98	98	98	-
**C**. *sBt***0.50**-*hAr***0.53**					
	Control	Test 1	Test 2	Test 3	Test 4
Toxin concentration	-	-	-	0.14 µg Cry1Ab/g fwt	57 ng Cry1Fa/mg total protein
%RefRandom	100	10	100	46	26
%*Bt*Random	100	100	90	55	22
GF50Bef	-	20	-	20	20
GF50Random	19	18	20	19	19
GF50After	-	-	18	18	18
Global GF50	20	20	20	20	20
%popdecrease (between generation 3 and 1)	63	63	63	63	63

We considered that the insect’s reproductive capacity remains stable whatever the native zone and the age of the adults at the time of the mating.

### 2.4. Observed %RefBef and %*Bt*After at Lower Toxin Concentration in the *Bt* Plants (*sBt* = 0.93 and *sBt* = 0.50) and Their Impacts on Resistance Management

The HD/R strategy requires *Bt* plants expressing high toxin concentration (*sBt* = 1) associated with a recessive resistance allele (*hAr* = 0). However, in our experiments, emergence asynchrony is significantly proportional to toxin concentration [13] (see Equations 4–7). Therefore, assortative mating could be limited by increasing toxin concentration in the *Bt* plants. However, toxin concentration, larval mortality and functional dominance are also linked [31,34,38,39]. Resistance dominance presents a plastic response depending on environmental parameters, with the recessivity of resistance being associated with more demanding environments [40]. For example, resistance to Cry1Ac in *P.gossypiella* is codominant at low concentrations (*sBt* 0.5; *hAr* = 0.53), partially recessive at intermediate concentrations (*sBt* = 0.93; *hAr* = 0.23), and completely recessive at high concentrations (*sBt* = 1, *hAr* = 0) [31]. 

We tested the impact of two toxin concentrations (*sBt* = 0.50, *i.e.*, 50% mortality of susceptible insects- and *sBt* = 0.93, *i.e.*, 93% mortality of susceptible insects-) with the associated dominances (*hAr* = 0.53 and *hAr* = 0.23, respectively) on the effectiveness of the HD/R. 

First, we varied emergence asynchrony (%*Bt*Random and %RefRandom: 0 to 100%, see Test 1 and Test 2 in Table 3) to assess the effectiveness of the HD/R strategy in relation to theoretical random mating disruption. Next, we used simulations to characterize the impact of the observed emergence asynchronies in relation to the Cry1Ab or Cry1Fa concentrations (see Test 3 and Test 4 in Table 3 and Equations 4–7). 

### 2.5. Impact of the Refuge Zone’s Relative Size

Emergence asynchrony potentially limits the effectiveness of the HD/R strategy. Moreover, this effectiveness is also reduced when *Bt* plants do not produce a high toxin concentration [26]. Here, we tested whether increasing the refuge zone (*v* = 5, 20 and 40%) could restore the effectiveness of the strategy. 

### 2.6. Model Output

The model investigated the effect of (i) random mating, (ii) dominance as linked to toxin concentration, and (iii) the relative size of the refuge, on resistance management in a pest population exposed to *Bt* plants. The most common indicator for comparing the spread of resistance in different simulations is the number of generations required to reach a 50% frequency of the resistance allele in the global population (GF50) [41,42]. This is a convenient measure of resistance, independent of any assumptions regarding population growth [43].

When emergence asynchrony occurred, the number of generations required to reach a 50% frequency of the resistance allele (GF50) in each group was also calculated: GF50Bef for the adults emerging from the refuge zone before those of the *Bt* zone; GF50After for the adults emerging from the *Bt* zone after those of the refuge zone and GF50Random for the adults simultaneously emerging from the refuge zone and the *Bt* zone. 

The indicator for population density was the percentage of population decrease between the first and the third generation. Faster to obtain (three generations are sufficient), this indicator is also easily interpretable because its sign (positive or negative) informs about changes in population density. Because it is a ratio, this indicator is independent of the initial size of the system. Our results therefore show how the system changes regardless of the area considered. 

%population decrease (between generation 3 and 1) = (1 − (density F3/density F1)) × 100(8)

## 3. Results and Discussion

The High Dose/Refuge Zone Strategy is based on the assumption that *Bt* plants express high toxin concentrations, and that enough susceptible insects mate with resistant insects, producing heterozygous offspring (ArAs), which will be completely purged in the *Bt* zone. However, the mating between susceptible and resistant insects could be rapidly disturbed if the choice of the sexual partner is influenced by physiology, morphology or behavior (*i.e.*, assortative mating). Assortative mating can be total or partial and modifies the genetic structure of the population only for the traits involved in the mating. A positive assortative mating favors crosses between individuals that are genotypically or phenotypically similar. In this case, there is a progressive increase of the homozygous genotype frequency and a corresponding decrease of the heterozygotes in the population. Therefore, preferential mating among resistant insects could disturb the effectiveness of the HD/R strategy [44]. According to our previous experimental results [13], a high toxin concentration slows insect development, inducing emergence asynchrony between individuals exposed and unexposed to the toxins. Similar conclusions have been made regarding *Ostrinia nubilalis*: a resistant population feeding on Cry1Ab-incorporated diet emerged approximately 7 days after susceptible *O. nubilalis* feeding on a control diet [19]. This favors the probability of intrazone matings between susceptible insects in the refuge zone and between resistant insects in the *Bt* zone and therefore disrupts random mating [24].

### 3.1. Effectiveness of Resistance Management with *Bt* Plants Expressing a High Toxin Concentration (*sBt* = 1) in Relation to Emergence Asynchrony

According to our simulations, the most efficient situation corresponds to the initial assumptions of the HD/R strategy: global random mating (%RefRandom = %*Bt*Random = 100%), *Bt* plants expressing a high toxin concentration (*sBt* = 1) eliminating 100% of the homozygote susceptible insects (AsAs) and a recessive resistance (*hAr* = 0). In this ideal context, the critical threshold of 50% resistance allele frequency in the population (GF50) is reached at the 48th generation, and there is a 99.7% population decrease between the first and the third generation (see Control in Table 3A). This is really the best situation for optimal control of both resistance and pest density. 

**Figure 2:**
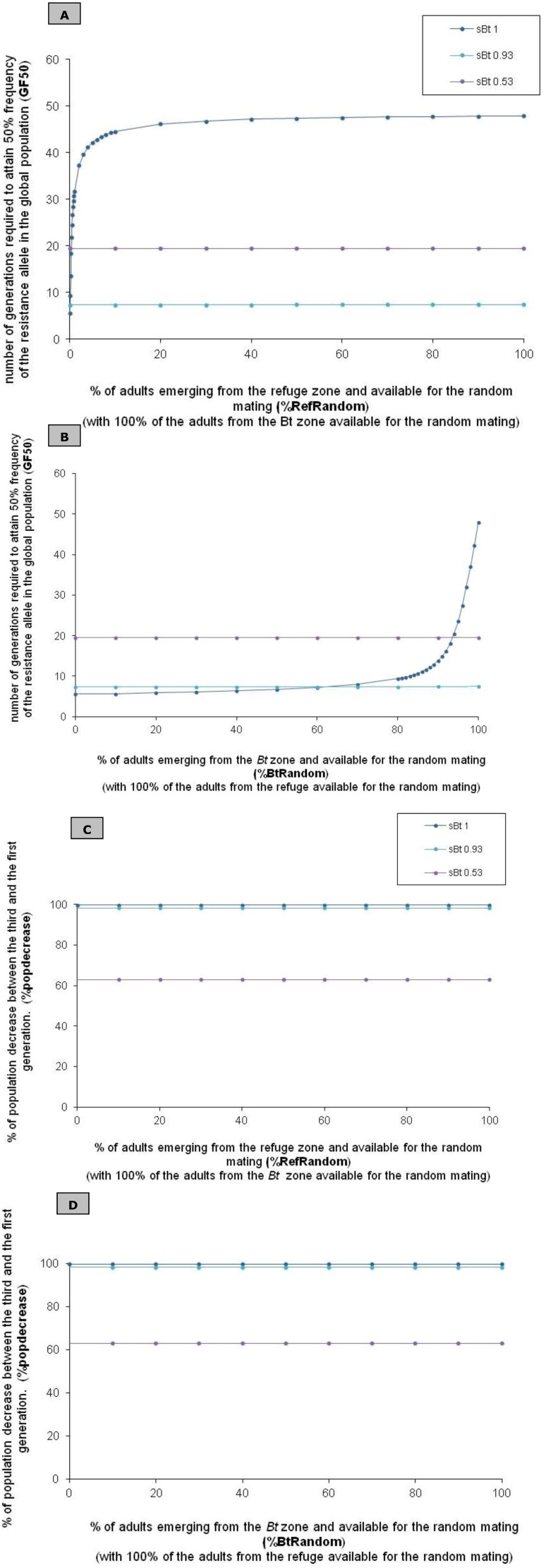
Resistance spread and population density in relation to emergence asynchrony and Cry toxin concentration based on a theoretical approach. (**A**) We tested the impact of emergence asynchrony in the refuge zone (%RefRandom) and of toxin concentration (*sBt*) on the resistance spread. The resistance spread is characterized by GF50 (number of generations required to reach a frequency of 50% of the resistance allele in the global population); (**B**) We tested the impact of emergence asynchrony in the *Bt* zone (%*Bt*Random) and of toxin concentration (*sBt*) on resistance spread, characterized by GF50; (**C**) We tested the impact of emergence asynchrony in the refuge zone (%RefRandom) and of toxin concentration (*sBt*) on population density. The indicator for population density is %popdecrease, the relative population decrease (in %) between the first and the third generation; (**D**) We tested the impact of emergence asynchrony in the *Bt* zone (%*Bt*Random) and of toxin concentration (*sBt*) on population density. The indicator for population density is %popdecrease.

When more than 10% of the adults of the refuge zone (%RefRandom ≥ 10) are involved in random mating with all of the adults emerging from the *Bt* zone (%*Bt*Random = 100%), the strategy is still efficient but slightly reduced (48 ≥ GF50 ≥ 45) (see Figure 2A and Test 1 in Table 3A). Below this 10% limit (%RefRandom < 10), the effectiveness of the HD/R strategy is highly reduced (e.g. 2%RefRandom: GF50: 37) (see Figure 2A).

On the contrary, even minor delays in adult emergence from the *Bt* zone highly reduce the effectiveness of the HD/R strategy (see Figure 2B). GF50 is reached after 14 generations if 90% of the adults emerging from the *Bt* zone are available for random mating with all of the adults of the refuge zone (90%*Bt*Random, 100%RefRandom) (see Figure 2Band Test 2 inTable 3A). The threshold is reached after one generation for the adults emerging from the *Bt* zone after the adults of the refuge zone. However, GF50 is reached after thirteen generations in the group composed by adults emerging simultaneously from the refuge and from the *Bt* zone, and GF50 corresponds to 14 generations in the total population. 

As recorded in our previous experiments [13], a high Cry1Ab concentration induces a complete asynchrony in emergence between the adults of the two zones and, there is thus no random mating between zones (100%RefBef and 100%*Bt*After) (see Test 3 in Table 3A). In this case, only one generation is necessary to reach GF50 in the group composed of adults emerging from the *Bt* zone. In the refuge zone and in the global system, GF50 is reached after six generations.

When *Bt* plants produce a high toxin concentration, the population decrease between the first and the third generation is constant whatever the asynchrony (99.7% popdecrease). 

In conclusion, temporal isolation and even minor delays could influence mating success and therefore the effectiveness of the refuge [19]. In our model, the effectiveness of the HD/R strategy is negatively influenced by emergence asynchrony, mainly by the late emergence of resistant insects from the *Bt* zone after emergence in the refuge zone. This temporally isolated group emerging from the *Bt* zone is only composed of homozygous resistant insects (ArAr) because all of the ArAs and AsAs are eliminated by ingestion of the high toxin concentration expressed by *Bt* plants (s*Bt* = 1 − *hAr* = 0). Thus, when emergence asynchrony is complete, resistance is reached in the group after one generation. Moreover, we observe that numerous adults emerging from the refuge zone carry one or two resistance alleles. Resistance appears in this non-*Bt* zone, where there is no selection pressure, because of the pollution by resistant alleles during random egg laying by resistant insects selected in the *Bt* zone. This “pollution” decreases the effectiveness of the refuge zone by diluting the resistance.

However, with *Bt* plants expressing a high toxin concentration, pest density control is not influenced by emergence asynchrony (see Table 3A). The effect of emergence asynchrony on population density is probably masked by the massive population decrease induced by the *Bt* zone because, during the first generation, few insects are able to survive on the large *Bt* zone. The impact of emergence asynchrony is thus not well detected and probably insignificant in comparison to mortality in the *Bt* zone. 

Two approaches are considered to reduce the negative impact of emergence asynchrony: reducing toxin concentration, which decreases emergence asynchrony and the recessivity of the resistance allele (see Section 3.2), or increasing the relative size of the refuge zone, which provides more susceptible alleles to dilute resistance for the next generation (see Section 3.3).

### 3.2. Reducing Toxin Concentration: Impact on the Effectiveness of Resistance Management

When mating is random (%RefRandom = %*Bt*Random = 100%), resistance is better controlled with *sBt* = 1 − *hAr* = 0 (GF50: 48) because all the ArAs are eliminated in the *Bt* zone (ArAs survival on *Bt* zone: 0% see Table 2), as recommended in the HD/R strategy. If *Bt* plants express lower toxin concentrations, the survival of susceptible homozygotes (AsAs) increases on the *Bt* zone (survival on *Bt* zone with *sBt* = 0.53: 76.5% ArAs and 50% AsAs; survival on *Bt* zone with *sBt* = 0.93: 28.39% ArAs and 0.07% AsAs). *sBt* = 0.53 is more efficient than *sBt* = 0.93 to control resistance (GF50: 20 *versus* GF50: 7, respectively) (see Table 2, control in Table 3B,C, respectively). 

Mortality induced by *Bt* plants influences population density control. With *sBt* = 0.93 − *hAr* = 0.23, there is a 98% population decrease between the first and the third generation and a 63% decrease with *sBt* = 0.50 − *hAr* = 0.53 (see Table 3B,C, respectively).

In contrast to the high toxin concentration associated with a recessive resistant allele (*sBt* = 1 − *hAr* = 0), emergence asynchrony does not influence the effectiveness of resistance management at lower selection pressures (GF50: 20 *sBt* = 0.53 and GF50:7 for *sBt* = 0.93, regardless of emergence asynchrony) (see Figure 2A,B, Table 3B,C respectively). The emergence asynchrony experimentally observed with two Cry toxin concentrations (Cry1Ab and Cry1Fa) has no impact on population control (see Figure 2C,D). (*sBt* = 0.53: 63% population decrease and *sBt* = 0.93: 98% population decrease; regardless of emergence asynchrony).

To conclude, emergence asynchrony has no impact on the effectiveness of the strategy nor on pest density control when *Bt* plants do not produce high toxin concentrations. The susceptible alleles are not fully eliminated from the *Bt* zone (some ArAs and AsAs survive) and these susceptible alleles sufficiently dilute resistance even if asynchrony is complete. Resistance control and density control are thus only dependent on toxin concentration and on the associated dominance of the resistance allele. Resistance is better controlled with *sBt* = 0.53 than with *sBt* = 0.93 because survival of insect carrying one or two resistance alleles is higher. More susceptible alleles are thus available to dilute resistance. However, the pest population is better controlled with *sBt* = 0.93 than with *sBt* = 0.53, because more susceptible insects (AsAs and ArAs) are eliminated from the *Bt* zone. As lower toxin concentrations are more favorable to pest population development in the *Bt* zone than high toxin concentrations, growers might not welcome our recommendation of using lower *Bt* concentrations, but such a strategy will help to reduce resistance development and thus has long-term benefit. 

### 3.3. Refuge Zone Relative Size: Impact on Resistance Management

#### 3.3.1. *sBt* = 1 − *hAr* = 0

**Figure 3:**
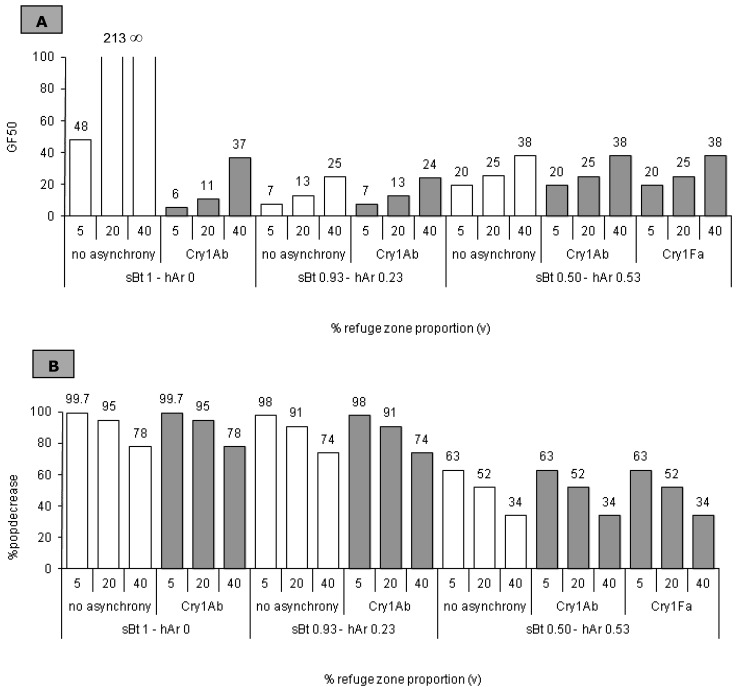
Resistance spread and population density in relation to emergence asynchrony, insect mortality (*sBt* = 1, 0.50, 0.93), resistance dominance (*hAr* = 0, 0.53, 0.23) and refuge zone relative size (*v* = 0.05, 0.2, 0.4), based on experimental results. (**A**) We tested the impact of emergence asynchrony on resistance spread. Emergence asynchrony was experimentally measured on different concentrations of Cry1Ab and Cry1Fa (*sBt*). Resistance spread is characterized by GF50 (number of generations required to reach a 50% frequency of the resistance allele in the global population); (**B**) We tested the impact of emergence asynchrony on population density. Emergence asynchrony was experimentally measured with different concentrations of Cry1Ab and Cry1Fa (*sBt*). The indicator for population density is %popdecrease the percentage of population decrease between the first and the third generation.

The negative impact of emergence asynchrony on HD/R effectiveness is partially compensated for by increasing the refuge zone (see Figure 3A). When asynchrony is complete, the larger refuge zone tested (40%) is not sufficient for the strategy to remain effective (GF50 control with 5% refuge: 48 generations; GF50 Cry1Ab with 40% refuge: 37 generations).

However, a large refuge zone reduces pest control (99.7% pop decrease at 4% refuge, 78% popdecrease at 40% refuge, regardless of the asynchrony) (see Figure 3B).

#### 3.3.2. *sBt* &lt; 1 − *hAr* &gt; 0

For the two values of *sBt*-*hAr* (*sBt* = 0.50 − *hAr* = 0.53 and *sBt* = 0.93 − *hAr* = 0.23), increasing refuge zone size enhances resistance control whatever the asynchrony in emergence (GF50: 7 for *sBt* = 0.93 and *v* = 5%; GF50: 13 for *sBt* = 0.93 and *v* = 20%; GF50: 20 for *sBt* = 0.50 and *v* = 5%; GF50: 25 for *sBt* = 50 and *v* = 20%) but reduces population control (98%popdecrease for *sBt* = 0.93 and *v* = 5%; 91%popdecrease for *sBt* = 0.93 and *v* 20%; 63%popdecrease for *sBt* = 0.50 and *v* = 5%; 52%popdecrease for *sBt* = 0.50 and *v* = 20%) (see Figure 3A,B).

As previously demonstrated [26], the effectiveness of the HD/R strategy increases in relation to the size of the refuge zone: the larger the refuge, the larger the population of susceptible insects in the global system and the more diluted the resistance. We notice that, as emergence asynchrony has no impact on the evolution of resistance when *Bt* plants do not produce a high toxin concentration, the increase of the refuge zone has a similar impact on resistance evolution regardless of the emergence asynchrony. 

However, a small refuge zone provides better pest control. Again, we have to take into account the compliance of the growers. Increasing the refuge zone could decrease the acceptability of this strategy by the growers unwilling to sacrifice a larger area of their field. 

In our model, we did not integrate any overlapping between generations. If the overlap is complete, there is a continuous supply of susceptible alleles, which would improve resistance management because resistance would always be diluted. 

The lengthening of insect development disrupts random mating but also influences the reproductive capacity of the insects emerging from the refuge zone. When the insects of the refuge zone emerge before the insects of the *Bt* zone, they are older when they mate with the latter. Many studies have demonstrated reduced mating success with increased age in Lepidoptera and Coleoptera: reduction of the number of matings of the females, reduction of the capacity to inseminate females, reduction of the number of transferred spermatophores, reduction of the number of eggs laid (fecundity) and reduction of egg viability [19,45,46,47].

At least six factors may contribute to reduced fecundity or fertility when mating is delayed: (1) a decrease in nutritional reserves available for egg development or oviposition; (2) a resorption of nutrients from the eggs before they are laid; (3) a reduced ability to store or transport viable sperm [48]; (4) a deterioration in viability of the eggs with time; (5) interference of oocyte degradation products with sperm migration and/or egg fertilization [49]; and (6) the laying of unfertilized eggs before mating.

Apart from the impact on assortative mating induced by the lengthening of development in the *Bt* zone reported in this paper, other causes could be involved in the disruption of random mating. 

First, random mating between the adults of the two zones is possible if all the emerging adults move to meet each other [44,50]. However, this precopulary dispersal can be more or less active in relation to the genotype and to the native zone. On non-*Bt* potato plants, resistant *Leptinotarsa decemlineata* engage in fewer flights than susceptible insects probably because of their reduced general fitness. On *Bt* potato plants, susceptible *Leptinotarsa decemlineata* do not develop flight muscles, which forbids flight and therefore random mating. The development of resistant insects on *Bt* potato plants delays the first flight but increases flight activity in comparison with insects reared on non-*Bt* potato plants. Such differences in precopulary flight between resistant and susceptible adults limit random mating between the two zones and the effectiveness of the HD/R strategy [51]. 

Secondly, random mating requires a similar probability of mating which is not always granted. For example, some degree of predispersal mating occurs in natural populations of *Ostrinia nubilalis*. At the local scale, resident females mate indifferently with resident or immigrant males, preserving random mating, but resident males rarely mate with immigrant females, which disturbs random mating and probably the effectiveness of the HD/R strategy. This avoidance of immigrant females could be due to factors such as older age, previous mating or reduced energy reserves [50].

Assortative mating could sometimes improve the effectiveness of the HD/R strategy if resistant pests are less attractive than susceptible individuals. This would confer them a reduced mating success relative to susceptible individuals, and would thus favor the restoration of susceptibility [51,52]. The resistant males of *Plutella xylostella* have a reduced success in performing successive matings. Faster loss of mating vigor in resistant males would be consistent with observed lower survival in resistant individuals [52]. Without competition for access to females, resistant and susceptible males of *Pectinophora gossypiella* had similar mating frequency. However, when competing for females, the resistant males that mated first sired significantly less offspring than susceptible males. This reduced first-male paternity in resistant males may involve reduced sperm precedence caused by mutation in a cadherin gene linked with resistance to *Bt* cotton [53].

## 4. Conclusions and Limitations

All models are inherently simplifications of a real-world situation. Although we acknowledge that a biologically accurate description of the system in question would require incorporation of enormous number of variables [54], we chose our model parameters parsimoniously, making several simplifications in order to facilitate and expedite analyses. Nonetheless, our model was sufficient to study the key changes in the system and to answer important questions about the impact of random mating disruption on the effectiveness of the HD/R strategy. 

## References

[1] Gallais A., Ricroch A. (2006). Plantes Transgéniques: Faits et Enjeux.

[2] Carlile W.R. (2006). Pesticide Selectivity, Health and the Environment.

[3] Metcalf R.L., Luckmann W.H. (1994). Introduction to Insect Pest Management.

[4] Lacey L., Goettel M. (1995). Current developments in microbial control of insect pests and prospects for the early 21st century. BioControl.

[5] Hofte H., Whiteley H.R. (1989). Insecticidal crystal proteins of *Bacillus thuringiensis*. Microbiol. Mol. Biol. Rev..

[6] Thacker J.R.M.D. (2002). An Introduction to Arthropod Pest Control.

[7] Joung K.-B., Côté J.-C. (2000). Une Analyse des Incidences Environnementales de L’insecticide Microbien Bacillus thuringiensis.

[8] James C. (2012). 2010 ISAAA Report on Global Status of Biotech/GM Crops. ISAAA Brief.

[9] Jorge F.-C. USDA Economic Research Service ERS/USDA Data—Adoption of Genetically Engineered Crops in the U.S. http://www.ers.usda.gov/data-products/adoption-of-genetically-engineered-crops-in-the-us.aspx/.

[10] Bates S.L., Zhao J.-Z., Roush R.T., Shelton A.M. (2005). Insect resistance management in GM crops: Past, present and future. Nat. Biotech..

[11] Caprio M., Summerford D., Simms S., Lacey L.A., Kaya H.K. (2000). Evaluating transgenic plants for suitability in pest and resistance management programs. Field Manual of Techniques in Invertebrate Pathology.

[12] Giles K.L., Hellmich R.L., Iverson C.T., Lewis L.C. (2000). Effects of transgenic *Bacillus thuringiensis* maize grain on *B. thuringiensis*-susceptible *Plodia interpunctella* (Lepidoptera: Pyralidae). J. Econ. Entomol..

[13] Gryspeirt A., Grégoire J.-C. (2012). Effects of Two Varieties of *Bacillus thuringiensis* Maize on the Biology of Plodia Interpunctella. Toxins.

[14] Sedlacek J.D., Komaravalli S.R., Hanley A.M., Price B.D., Davis P.M. (2001). Life History Attributes of Indian Meal Moth (Lepidoptera: Pyralidae) and Angoumois Grain Moth (Lepidoptera: Gelechiidae) Reared on Transgenic Corn Kernels. J. Econ. Entomol..

[15] Liu Y.-B., Tabashnik B.E., Dennehy T.J., Patin A.L., Bartlett A.C. (1999). Development time and resistance to *Bt* crops. Nature.

[16] Liu Y.-B., Tabashnik B.E., Dennehy T.J., Patin A.L., Sims M.A., Meyer S.K., Carrière Y. (2001). Effects of *Bt* Cotton and Cry1Ac Toxin on Survival and Development of Pink Bollworm (Lepidoptera: Gelechiidae). J. Econ. Entomol..

[17] Khalique F., Ahmed K. (2003). Impact of *Bacillus thuringiensis* subsp. kurstaki on biology of *Helicoverpa zea armigera*. Pakistan J. Biol. Sci..

[18] Bird L.J., Akhurst R.J. (2004). Relative fitness of Cry1A-resistant and -susceptible *Helicoverpa armigera* (Lepidoptera: Noctuidae) on conventional and transgenic cotton. J. Econ. Entomol..

[19] Lopez M.D., Sumerford D.V., Lewis L.C. (2010). Nosema pyrausta and Cry1Ab-incorporated diet led to decreased survival and developmental delays in European corn borer. Entomologia Experimentalis et Applicata.

[20] Horner T.A., Dively G.P., Herbert D.A. (2003). Development, survival and fitness performance of *Helicoverpa zea* (Lepidoptera: Noctuidae) in MON810 *Bt* field corn. J. Econ. Entomol..

[21] Storer N.P., Van Duyn J.W., Kennedy G.G. (2001). Life history traits of *Helicoverpa zea* (Lepidoptera: Noctuidae) on non-*Bt* and *Bt* transgenic corn hybrids in eastern North Carolina. J. Econ. Entomol..

[22] Caprio M.A. (1998). Evaluating Resistance Management Strategies for Multiple Toxins in the Presence of External Refuges. J. Econ. Entomol..

[23] Horner T.A., Dively G.P., Herbert D.A. (2003). Effects of MON810 *Bt* field corn on adult emergence of *Helicoverpa zea* (Lepidoptera: Noctuidae). J. Econ. Entomol..

[24] Peck S.L., Gould F., Ellner S.P. (1999). Spread of resistance in spatially extended regions of transgenic cotton: Implications for management of *Heliothis virescens* (Lepidoptera: Noctuidae). J. Econ. Entomol..

[25] Fabrick J.A., Forlow Jech L., Henneberry T.J. (2009). Novel pink bollworm resistance to the *Bt* toxin Cry 1Ac: Effects on mating, oviposition, larval development and survival. J. Insect Sci..

[26] Gryspeirt A., Grégoire J.-C. (2012). Effectiveness of the High Dose/Refuge Strategy for Managing Pest Resistance to *Bacillus thuringiensis* (*Bt*) Plants Expressing One or Two Toxins. Toxins.

[27] US EPA Biopesticides Registration Action Document—Bacillus thuringiensis Plant-Incorporated Protectants| Pesticides| US EPA. http://www.epa.gov/oppbppd1/biopesticides/pips/bt_brad.htm.

[28] Guse C.A., Onstad D.W., Buschman L.L., Porter P., Higgins R.A., Sloderbeck P.E., Cronholm G.B., Peairs F.B. (2002). Modeling the development of resistance by stalk-boring Lepidoptera (Crambidae) in areas with irrigated transgenic corn. Environ. Entomol..

[29] Andow D.A., Olson D.M., Hellmich R.L., Alstad D.N., Hutchison W.D. (2000). Frequency of resistance to *Bacillus thuringiensis* toxin Cry1Ab in an Iowa population of European corn borer (Lepidoptera: Crambidae). J. Econ. Entomol..

[30] Tabashnik B.E., Patin A.L., Dennehy T.J., Liu Y.B., Carrière Y., Sims M.A., Antilla L. (2000). Frequency of resistance to *Bacillus thuringiensis* in field populations of pink bollworm. Proc. Nat. Acad. Sci. USA.

[31] Liu Y.B., Tabashnik B.E., Meyer S.K., Carrière Y., Bartlett A.C. (2001). Genetics of pink bollworm resistance to *Bacillus thuringiensis* toxin Cry1Ac. J. Econ. Entomol..

[32] Vacher C., Bourguet D., Rousset F., Chevillon C., Hochberg M.E. (2003). Modelling the spatial configuration of refuges for a sustainable control of pests: A case study of *Bt* cotton. J. Evol. Biol..

[33] Sayyed A.H., Wright D.J. (2001). Fitness costs and stability of resistance to *Bacillus thuringiensis* in a field population of the diamondback moth *Plutella xylostella* L. Ecol. Entomol..

[34] Gould F., Anderson A., Reynolds A., Bumgarner L., Moar W. (1995). Selection and genetic analysis of a *Heliothis virescens* (Lepidoptera: Noctuidae) strain with high levels of resistance to *Bacillus thuringiensis* toxins. J. Econ. Entomol..

[35] Gassmann A.J., Carrière Y., Tabashnik B.E. (2009). Fitness costs of insect resistance to *Bacillus thuringiensis*. Annu. Rev. Entomol..

[36] Lenormand T., Raymond M. (1998). Resistance management: The stable zone strategy. Proc. Biol. Sci..

[37] Conner J.K., Hartl D.L. (2004). A Primer of Ecological Genetics.

[38] Sayyed A.H., Haward R., Herrero S., Ferre J., Wright D.J. (2000). Genetic and Biochemical Approach for Characterization of Resistance to *Bacillus thuringiensis* Toxin Cry1Ac in a Field Population of the Diamondback Moth, *Plutella xylostella*. Appl. Environ. Microbiol..

[39] Rawlings P., Davidson G., Sakai R.K., Rathor H.R., Aslamkhan M., Curtis C.F. (1981). Field measurement of the effective dominance of an insecticide resistance in anopheline mosquitos. Bull. World Health Organ.

[40] Bourguet D., Prout M., Raymond M. (1996). Dominance of insecticide resistance presents a plastic response. Genetics.

[41] Mallet J., Porter P. (1992). Preventing insect adaptation to insect-resistant crops: Are seed mixtures or refugia the best strategy?. Proc. Biol. Sci..

[42] Georghiou G.P., Taylor C.E. (1977). Genetic and biological influences in the evolution of insecticide resistance. J. Econ. Entomol..

[43] Roush R.T. (1998). Two-toxin strategies for management of insecticidal transgenic crops: Can pyramiding succeed where pesticide mixtures have not?. Phil. Trans. R Soc. Lond. B Biol. Sci..

[44] Lenormand T. (2002). Gene flow and the limits to natural selection. Trends Ecol. Evol..

[45] Lingren P.D., Warner W.B., Henneberry T.J. (1988). Influence of Delayed Mating on Egg-Production, Egg Viability, Mating, and Longevity of Female Pink-Bollworm (Lepidoptera, Gelechiidae). Environ. Entomol..

[46] Huang F., Subramanyam B. (2003). Effects of delayed mating on reproductive performance of *Plodia interpunctella* (H\übner)(Lepidoptera: Pyralidae). J. Stored Prod. Res..

[47] Wenninger E.J., Averill A.L. (2006). Effects of delayed mating on reproductive output of female oriental beetle *Anomala orientalis* (Coleoptera: Scarabaeidae). Agric. Forest Entomol..

[48] Proshold F.I. (1996). Reproductive capacity of laboratory-reared gypsy moths (Lepidoptera: Lymantriidae): Effect of age of female at time of mating. J. Econ. Entomol..

[49] Torres-Vila L.M., Rodríguez-Molina M.C., Stockel J. (2002). Delayed mating reduces reproductive output of female European grapevine moth, *Lobesia botrana* (Lepidoptera: Tortricidae). Bull. Entomol. Res..

[50] Dalecky A., Ponsard S., Bailey R.I., Pélissier C., Bourguet D. (2006). Resistance evolution to *Bt* crops: Predispersal mating of European corn borers. PLoS Biol..

[51] Alyokhin A., Ferro D. (1999). Relative fitness of Colorado potato beetle (Coleoptera: Chrysomelidae) resistant and susceptible to the *Bacillus thuringiensis* Cry3A toxin. J. Econ. Entomol..

[52] Groeters F.R., Tabashnik B.E., Finson N., Johnson M.W. (1993). Resistance to *Bacillus thuringiensis* affects mating success of the diamondback moth (Lepidoptera: Plutellidae). J. Econ. Entomol..

[53] Higginson D.M., Morin S., Nyboer M.E., Biggs R.W., Tabashnik B.E., Carrière Y. (2005). Evolutionary trade-offs of insect resistance to *Bacillus thuringiensis* crops: Fitness cost affecting paternity. Evolution.

[54] Mangel M., Clark C.W. (1988). Dynamic Modeling in Behavioral Ecology.

